# The sinusoidal probe: a new approach to improve electrode longevity

**DOI:** 10.3389/fneng.2014.00010

**Published:** 2014-04-29

**Authors:** Harbaljit S. Sohal, Andrew Jackson, Richard Jackson, Gavin J. Clowry, Konstantin Vassilevski, Anthony O’Neill, Stuart N. Baker

**Affiliations:** ^1^Newcastle Movement Lab, Institute of Neuroscience, Newcastle UniversityNewcastle Upon Tyne, UK; ^2^School of Electrical and Electronic Engineering, Newcastle UniversityNewcastle Upon Tyne, UK

**Keywords:** flexible, microelectrode, chronic, electrophysiology, gliosis, long term, electrode

## Abstract

Micromotion between the brain and implanted electrodes is a major contributor to the failure of invasive brain–machine interfaces. Movements of the electrode tip cause recording instabilities while spike amplitudes decline over the weeks/months post-implantation due to glial cell activation caused by sustained mechanical trauma. We have designed a sinusoidal probe in order to reduce movement of the recording tip relative to the surrounding neural tissue. The probe was microfabricated from flexible materials and incorporated a sinusoidal shaft to minimize tethering forces and a 3D spheroid tip to anchor the recording site within the brain. Compared to standard microwire electrodes, the signal-to-noise ratio and local field potential power of sinusoidal probe recordings from rabbits was more stable across recording periods up to 678 days. Histological quantification of microglia and astrocytes showed reduced neuronal tissue damage especially for the tip region between 6 and 24 months post-implantation. We suggest that the micromotion-reducing measures incorporated into our design, at least partially, decreased the magnitude of gliosis, resulting in enhanced longevity of recording.

## INTRODUCTION

Brain–machine-interface (BMIs) decode motor information from cortical activity, providing control of computers, assistive devices or functional electrical stimulation to restore function after paralysis (Nicolelis, 2001; Taylor et al., 2002; Schwartz, 2004; Hochberg et al., 2006, 2012; Bartels et al., 2008; Zimmermann et al., 2011; Collinger et al., 2012). Invasive BMIs use intracortical microelectrodes to record from neurons in close proximity (~100–150 μm) to the recording tip (Biran et al., 2005, 2007; Polikov et al., 2005; Perge et al., 2013). Unfortunately, sustained immune activation caused by the foreign body response with time leads to neuronal loss around microelectrodes (Polikov et al., 2005). Furthermore, immune mediators such as astrocytes and microglia ensheath electrode recording sites, isolating the electrode from surrounding brain tissue and releasing detrimental immune factors that compromise neuronal function (Biran et al., 2005; Polikov et al., 2005; Winslow et al., 2010; Winslow and Tresco, 2010; Collinger et al., 2012). These mechanisms cause instabilities in the recordings obtained from both microwire and silicon probes (Jackson and Fetz, 2007; Dickey et al., 2009) and can lead to device failure within weeks or months after chronic implantation (Polikov et al., 2005; Ward et al., 2009; Collinger et al., 2012).

An important contributor to the sustained tissue response is the modulus mismatch between the brain and implanted microelectrodes (Kozai and Kipke, 2009). Typical electrode materials include silicon (e.g., the “Utah” array, Rousche and Normann, 1998 and “Michigan” probe, Kipke et al., 2003) and metal microwires (Nicolelis et al., 2003; Jackson and Fetz, 2007) which have a higher Young’s modulus (YM, ~150 GPa) than brain tissue (5–30 KPa; Miller et al., 2000; Potter et al., 2012). This mismatch means microelectrodes anchored to the skull cannot accommodate movement of the brain within the cranial cavity (Biran et al., 2007; Thelin et al., 2011). Even if electrodes are designed to float with the brain, deformation of the tissue will change the position of deep neurons relative to an anchoring point at the brain surface (Biran et al., 2005; LaPlaca et al., 2005; Subbaroyan et al., 2005; Gilletti and Muthuswamy, 2006; Kozai and Kipke, 2009; Yoshida Kozai et al., 2012; Hwang and Andersen, 2013; Kim et al., 2013). Damage caused by electrode movement relative to surrounding tissue will be exacerbated by the sharp tip profiles typically used to aid insertion. The amount of damage around the tips can be estimated through cell loss over time for current electrode arrays. For the Utah array, 61% of cells that were recorded on the first recording session were lost after 2 weeks (Dickey et al., 2009). Similar neuronal loss has been shown for microwire electrodes (Nicolelis et al., 2003; Jackson and Fetz, 2007). Microelectrodes can record between 100 and 150 μm away from their recording sites (Polikov et al., 2005; Seymour et al., 2011). For a single electrode shank, this equates to a 6750 mm^3^ (150 × 150 × 300 μm) volume around the electrode. For the human brain there are an estimated 6.66 × 10^4^ neurons per mm^3^ (Allen et al., 2005; Azevedo et al., 2009; Marblestone et al., 2013). Through extrapolation, if an estimated 61% of cells are lost for every two weeks of recording, then just a solitary cell will remain after ~40 weeks post-implant around a single shank, assuming the electrode can record from every cell in that given brain volume and neurons are lost linearly over time.

Micromotion can be reduced using flexible materials (Polikov et al., 2005; Kozai and Kipke, 2009; Harris et al., 2011) or by decreasing the overall footprint of the microelectrode (Yoshida Kozai et al., 2012; Suyatin et al., 2013). Flexible arrays require specialized insertion techniques to penetrate the dura or pia. Proposed methods include temporary stiffening with biodegradable solutions (Chorover and DeLuca, 1972; Kohler et al., 2009; Witteveen et al., 2010; Hassler et al., 2011) or soluble polymers such as polyethylene glycol (PEG; Takeuchi et al., 2004) prior to insertion. Some flexible arrays have sharp anchoring structures to restrict further the movement of the recording site relative to the surrounding neuronal tissue (Kohler et al., 2009; Wu et al., 2011). Nevertheless, while flexible materials can bend to shorten the distance between the anchoring point and the tip, a straight electrode, no matter how flexible, cannot accommodate deformations of the brain that increase this distance.

All current chronic electrode designs, which have evolved from the straight shafts traditionally used for acute recording share this fundamental limitation. We propose a simple yet radical solution: a novel flexible electrode which is not straight and can therefore lengthen and shorten to accommodate brain deformations. Specifically, we designed an electrode shaft with a sinusoidal profile mechanically to decouple the recording site from the fixed end of the electrode. Moreover, we incorporated a spherical tip to anchor the recording sites relative to the surrounding tissue and minimize damage. The novel electrode was microfabricated and tested *in vivo* over a 6–24 month period in rabbits. The electrode was compared to conventional rigid microwires of the same recording tip diameter. Microwires were chosen as a comparison as they have been well characterized in many species in chronic studies (Nicolelis et al., 2003; Jackson and Fetz, 2007; Winslow et al., 2010) and have reduced gliosis responses compared to standard silicon probes (Winslow et al., 2010; Yoshida Kozai et al., 2012).

The electrophysiological recordings and post-mortem characterization of the tissue response compared to conventional microwires suggest that the novel design features incorporated in our sinusoidal electrode may improve the long-term stability of recordings for BMI applications.

## MATERIALS AND METHODS

### SINUSOIDAL PROBE: DESIGN AND MICROFABRICATION

The probe consisted of three W/Ti (80/20 wt %) recording sites encased in parylene-C. Tungsten is a good recording metal (Hubel, 1959). However, with a YM of 400 GPa, it is not flexible compared to other metals (Geddes and Roeder, 2003). Therefore, tungsten was sintered with titanium to increase flexibility, resulting in a YM of ~200 GPa. Parylene-C was chosen as it is biocompatible, flexible, and a FDA-approved dielectric for biomedical applications (Seymour and Kipke, 2006; Kozai et al., 2010; Winslow et al., 2010; Yoshida Kozai et al., 2012).

**FIGURE 1:**
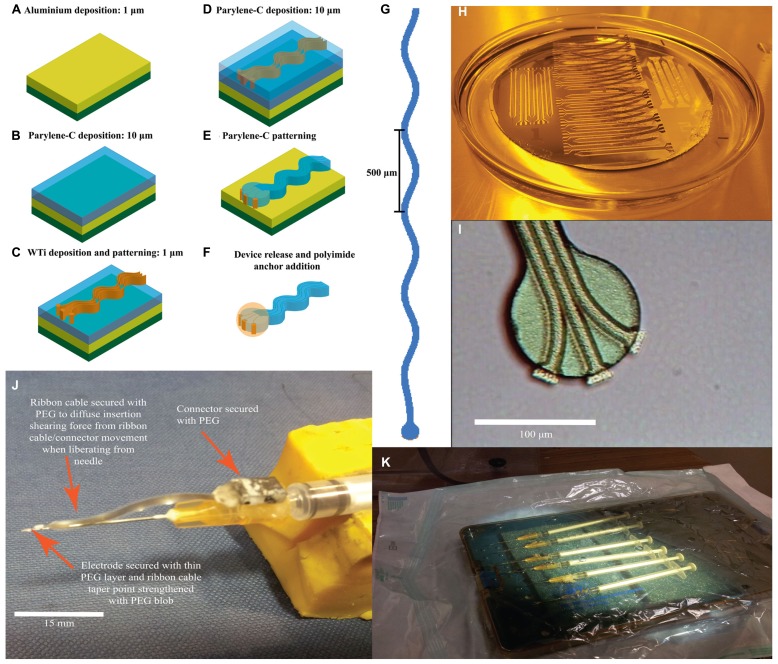
**Sinusoidal probe microfabrication and surgery preparation. (A–F)** Sequential microfabrication steps for the sinusoidal probe concentrating on the recording end (illustrative and not to scale): **(A)** 1 μm aluminum ebeam deposition as a sacrificial layer on a silicon substrate. **(B)** Parylene-C deposition through a CVD process. **(C)** WTi sputter deposition and patterning to define electrode tracks, bondpad and recording site regions. **(D)** Second layer parylene-C deposition through CVD. **(E)** Patterning of both parylene-C layers using oxygen plasma. **(F)** Device release using a TMAH based photoresist developer and addition of 3D polyimide ball anchor. **(G)** Mask layout for the 3 mm version of the probe. Three metal tracks ran within an electrode shaft that was 20 μm deep, 35 μm wide and had a sinusoidal profile with cycles of 100 μm amplitude and 500 μm period. **(H)** Successful device release in TMAH. **(I)** Optical microscopy image of the recording end showing three separate and isolated electrode recording sites, pre-polyimide anchor addition. The protruding electrode recording sites may appear thicker due to the underlying first parylene-C layer, which is not removed during WTi etching. **(J)** Attached sinusoidal probe to improvised insertion carrier to allow for successful brain penetration and electrode insertion. **(K)** Electrodes under sterile conditions, placed in an autoclaved container and then into surgical bag primed for UV light sterilization.

The recording end of the electrode was a parylene disk of 100 μm diameter, with three protruding, exposed recording sites to aid single-unit isolation (Gray et al., 1995). Three metal tracks ran within an electrode shaft that was 20 μm deep, 35 μm wide and had a sinusoidal profile with cycles of 100 μm amplitude and 500 μm period. We report data from two design iterations, which differed in length and how connection to the electrode was made. The first generation electrode was 5.5 mm in length and had a bondpad (approximately 400 μm^2^) at the end of the sinusoidal shaft, to which individual wires were attached. The second generation electrode was 3 mm in length (**Figure 1G**) and incorporated an integrated ribbon cable (3 cm long and 3 mm wide) leading to a standard connector (micro ps1/ps2 series, Omnetics Connector Corporation, USA).

Fabrication began with a three-inch silicon wafer (Compart, UK) onto which a 1 μm aluminum sacrificial layer was e-beam deposited (BOC-Edwards auto ebeam evaporator, UK) to increase adhesion between the first polymeric layer during the fabrication process and aid device release (**Figure 1A**). The first layer of parylene-C was deposited with a thickness up to 10 μm, through a chemical vapor deposition process (CVD; **Figure 1B**). Next, a 1 μm WTi layer (80/20 wt%) was magnetron sputter deposited (Kurt J. Lesker PVD 75 vacuum deposition, USA) with a power setting of 100 W (**Figure 1C**). After masking with AZ-5214e photoresist (HD microchemical, Germany), the metal layer was patterned with SF_6_ reactive ion etch (RIE; Plasma-Therm 790, USA) using parameters of 200 W, 150 mTorr, and 10 sccm flow rate to define the overall electrode tracks, bondpad and recording site regions (**Figure 1C**). A slight over-etch was employed to surface roughen the first parylene-C layer to aid adhesion of the second layer, again deposited through CVD (**Figure 1D**). Subsequently both parylene-C layers were patterned using oxygen RIE (Plasma-Therm 790, USA) with parameters of 200 W, 50 mTorr, and 18 sccm flow rate to form the overall electrode shape and to re-open recording site/bond pad regions (**Figure 1E**). A 40 nm thick titanium mask was e-beamed deposited and patterned (Kurt J. Lesker PVD 75 vacuum deposition) for this process. Exposed WTi was unaffected by parylene-C oxygen RIE. Finally, the electrodes were released from the silicon substrate by etching the aluminum sacrificial layer using 3% tetramethylammonia hydroxide (TMAH) as found in AZ-326 MIF photoresist developer (HD MicroChemicals, Germany; **Figures 1E,H**). Post-release (**Figure 1F**), the recording end had three clearly isolated electrode recording sites (**Figure 1I**).

Connectors were attached to the bondpad region using conductive paint (RS Components, UK) and insulated with two-part epoxy (Araldite: Farnell, UK). A polyimide ball was added by dipping the recording end first in VM 652 adhesion promoter (HD Microchemicals, Germany) and then in PI-5878G (HD Microchemicals, Germany) forming a small sphere around the electrode tip. The added sphere had a diameter of less than 100 μm as confirmed through optical microscopy during fabrication and further confirmed through post-hoc histology via the implantation site size. The polyimide was soft-baked at 120°C for 30 min in a convection oven. To re-open the covered recording sites, the electrode tip was dipped into TMAH for a few seconds. Electrode tip exposure was confirmed by tip impedances (at 1 kHz in saline) comparable to pre-polyimide application.

To aid insertion in to the brain, we used carriers made from 0.229 mm diameter sharpened stainless steel electrodes (Microprobe INC., USA) emerging from 25 g hypodermic needles (**Figure 1J**). Electrodes and carriers were cleaned with ethanol before being attached with PEG and sterilized with ultra-violet irradiation (**Figure 1K**).

### ANIMALS AND SURGICAL PROCEDURES

All experiments were approved by the local ethics committee at Newcastle University and were performed under appropriate Home Office licenses in accordance with the UK Animals (Scientific Procedures) Act 1986. Electrodes were implanted into the sensorimotor representation of five New Zealand white rabbits (*Oryctolagus cuniculus*; Subjects R, J, N, L, Lo). After a midline skin incision and burr-hole craniotomies, electrodes were inserted stereotaxically at coordinates relative to bregma of 4 mm anterior to 4 mm posterior and 0.5 to 7 mm lateral (Gould, 1986; Swadlow, 1989, 1990, 1994; Swadlow and Hicks, 1996). Electrodes were inserted quickly (Bjornsson et al., 2006) using the stereotaxic manipulator. The PEG was dissolved with warm saline to release the electrode from the carrier, which was subsequently removed. Microwire electrodes (50 μm diameter Teflon-insulated tungsten) were inserted manually with surgical forceps. Connectors were attached to the skull with skull screws and dental cement. A wire wrapped around one skull screw served as ground and reference for the recordings. In this study, 4–5 sinusoidal electrodes were inserted per animal for both the first generation (Rabbit N, L) and second generation (Rabbit- R, J, Lo). Between 2 and 4 microwire electrodes were also inserted for comparison in each animal.

### ELECTROPHYSIOLOGY DATA ACQUISITION

Tri-weekly recording sessions began one week post-surgery. Animals were individually placed in a custom-made restraining box while neuronal activity was amplified (10× MPA8I headstage followed by 1000× PGA1632 amplifier; Multichannel systems, Germany) and recorded (Power 1401, Cambridge Electronic Design, UK). Spike channels were filtered between 0.3 and 8 kHz and sampled at 25 kHz while local field potentials (LFP) were filtered between 1 and 300 Hz and sampled at 1 kHz.

### CHRONIC RECORDING PERFORMANCE EVALUATION

In this study, statistical comparisons combined data across all electrodes of the same type implanted in each animal.

We analyzed LFP, spike activity and high voltage spindle responses (HVS) from both sinusoidal and microwire electrodes. The HVS activity is characterized by highly synchronous spike and wave patterns, typically only seen when the animal remains motionless with eyes open (Buzsáki et al., 1988).

All analysis was performed within the Matlab environment (version 2009a, MathWorks, USA). LFP power spectra for each electrode were constructed using a fast-Fourier transform and the spectrum was divided into distinct bands. The mean power in each band was calculated for each session across the chronic recording period.

For HVS responses and spiking, principle component analysis spike sorting was performed with custom written software GetSpike. This enabled spikes to be sorted by initial thresholding and cluster cutting with times recorded. Accepted waveforms consisted of sections five sampling points before and 15 after the threshold crossing. The mean peak-to-peak amplitude, noise and signal-to-noise ratio (SNR) were evaluated daily across the chronic period. The SNR was calculated in accordance to Suner et al. (2005). For the signal, the peak-to-peak amplitude (A) of the mean waveform was calculated. For the noise, the mean waveform was subtracted from all waveforms, with the standard deviation calculated from the resulting values. Noise was then calculated as 2× the average standard deviation (ε). SNR was then calculated as A/ε. For statistical analysis to compare recorded responses, percentile-bootstrapping was used because of unequal sample sizes: each sinusoidal probe had three recording sites compared to one microwire recording site. Spike events from the full dataset were drawn randomly (with replacement) to create surrogate datasets with the same number of events as the real data, but distributed evenly between sinusoidal and microwire electrodes. This procedure was repeated 10000 times to estimate the expected difference in each measure under the null hypothesis of no difference between electrode types. The null hypothesis was rejected if the observed difference fell outside the 2.5–97.5% percentile range of the bootstrapped distribution.

### HISTOLOGY ANALYSIS

Post-mortem histology was used to assess tissue response to implanted electrodes. After anesthesia was induced with hypnorm (0.3 ml/kg i.m.) and midazolam (2 mg/kg i.v.), animals were perfused transcardially with phosphate buffered saline (PBS) and then formal saline (10%). The relevant brain regions were removed and transferred to incrementally increasing sucrose solutions (10–30%) for cryoprotection before cutting into 50 μm sections perpendicular to the electrode tracks.

The tissue was stained for microglia, astrocytes, and neurofilament. Slices were incubated with 3% normal horse serum (Vector Labs, UK: S-2000) to prevent non-specific binding, and incubated overnight with relevant concentrations of the primary antibodies GFAP (1:500, Sigma-Aldrich, UK), isolectin-b4 [1:200, Vector Labs, UK: Biotinylated Griffonia (Bandeiraea) Simplicifolia Lectin 1] and where appropriate, SMI-32 (1:1000, Cambridge Biosciences, UK: R-500) diluted in PBS at 4°C. GFAP and Neurofilament slices underwent incubation with biotinylated antimouse for 2 h. All slices were then incubated with horseradish peroxide streptavidin (Vector Labs, UK: SA-5004) for 1 h.

Finally, the diaminobenzidine (DAB) reaction was performed (Sigma–Aldrich, UK). Slices were incubated with DAB and placed in PBS after the reaction. Slices were dehydrated in an ascending alcohol series (5 min of 70, 95, 100, and 100% ethanol) and 2, 10 min histoclear washes (Sigma-Aldrich, UK), before cover slips were mounted with histomount (Sigma-Aldrich, UK).

Images were taken at 10× magnification at a pre-saturation exposure time dependent on the staining using axiovision software (Carl Zeiss Microimaging, Germany) before normalization using custom Matlab software. Background levels were assessed from tissue at least 1 mm away from the electrode implantation site, and subtracted from electrode tract images to leave the glial response around electrodes which was inverted (using the “imcomplement” function on Matlab) and normalized by the background level. Images were imported into ImageJ64 software (NIH, USA) to determine the radial distribution of staining intensity around electrode tracks. This analysis was carried out on all sections stained for microglia, astrocytes and where appropriate neurofilament. To compare the overall differences between sinusoidal and microwire electrodes, the mean of at least 15 tracks were compared between each electrode type.

Graphs were plotted of mean intensity as a function of radial distance from each electrodes type. Paired *t*-tests were performed for distances between 50 and 500 μm away, with a significance level of *P* < 0.05, in accordance with previous literature (Biran et al., 2005; Winslow et al., 2010; Winslow and Tresco, 2010; Potter et al., 2012). In addition, we assessed the Bonferroni-corrected significance of the smallest *P* value for each depth.

## RESULTS

### ELECTRODE IMPEDANCE

All sinusoidal probes had pre-implantation impedances less than the 4–5 MΩ considered the limit for single unit recordings (Ludwig et al., 2006, 2011). The mean (±SEM) impedance at 1 kHz was 559 ± 88 kΩ (*n* = 99), compared with 200–400 kΩ for microwires.

### ELECTROPHYSIOLOGY: LFP RECORDINGS

**FIGURE 2:**
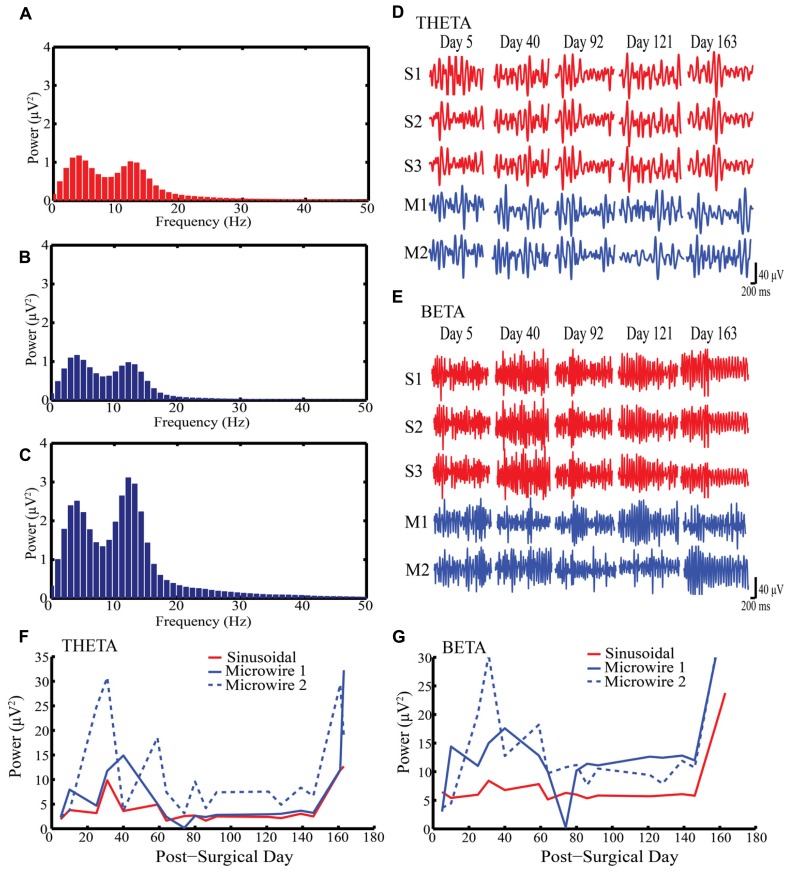
**Local field potential (LFP) recordings obtained from Rabbit-J over a 163 day indwelling period. (A–C)** Example power spectra for one sinusoidal and two microwire electrodes respectively showing peaks at both theta and beta bands **(D)** Example of filtered recordings from selected days for theta band from all electrodes, S1–3 are the three individual electrode recording sites on the sinusoidal probe, M1–2 are two separate microwire electrodes. **(E)** Example of filtered recordings from selected days for beta band from all electrodes. **(F,G)** Daily recorded power over the indwelling period for both theta and beta activity, respectively. The same LFP signal was captured by all three electrode recording sites for the sinusoidal probe over the indwelling period. Note the stability in LFP power for the sinusoidal compared to the microwire electrode.

**Figures 2A–C** shows example LFP power spectra for sinusoidal and microwire electrodes (Rabbit-J, second generation probe). Two clear oscillatory bands can be seen: theta (4–8 Hz) and beta (13–30 Hz) which were separated by band-pass filtering (**Figures 2D,E**). In this animal, LFP recordings were obtained from one sinusoidal and two microwire electrodes over a 163 day period (**Figures 2A–C**). The LFP signal obtained from each of the three recording sites of the sinusoidal electrode were very similar (*R*^2^ = 0.98, **Figures 2D,E**). Although the microwire electrode had higher LFP power values for both theta and beta frequency bands, the sinusoidal electrode recording was more stable over time (**Figures 2F,G**). Across the duration of the implant period, the standard deviation of the recorded power was 3.5, 7.8, and 9.9 μV^2^ (theta frequency) and 5.5, 11.2, and 11.6 μV^2^ (beta frequency) for the one sinusoidal and two microwire electrodes, respectively. Lower standard deviation values, measured across the recording sessions on different days, indicated that the LFP signal obtained for the sinusoidal probe appeared more stable across the chronic recording period in this animal than the comparator microwires. This result was also replicated for electrodes implanted in rabbit-R.

### ELECTROPHYSIOLOGY: HIGH VOLTAGE SPINDLE RESPONSES

**FIGURE 3:**
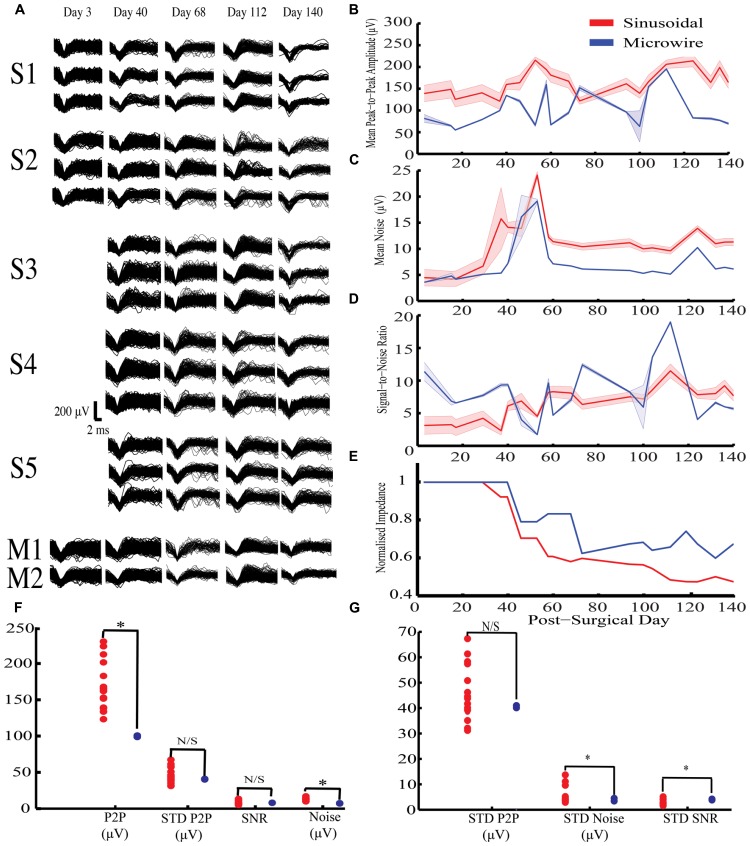
**Chronic high voltage spindle (HVS) responses from both sinusoidal and microwire electrode over a 140 day indwelling period from rabbit-R. (A)** Selected overlain waveforms over the recording period, S1–5 are five different sinusoidal probes and M1–2 are two different microwire electrodes.**(B–E)** Overall sinusoidal (*n* = 15 recording sites) and microwire (*n* = 2 recording sites) chronic performance over a 140 day indwelling period (mean ± SEM): **(B)** Mean peak to peak amplitude recorded per recording session. **(C)** Average noise amplitude recorded per recording session. **(D)** Signal-to-noise ratio over the entire recording period. **(E)** Impedance normalized to the first recording session. There was an overall decrease in impedance for the sinusoidal (*R*^2^ = 0.84) and microwire (*R*^2^ = 0.78) electrode over the indwelling period. **(F)** Statistical comparison of daily obtained recording parameters using bootstrapping methods between the two electrode types. **(G)** Comparisons of standard deviation values obtained across the entire recording period to analyze overall recording parameter stability using bootstrapping methods between the two electrode types. P2P = peak-to-peak amplitude. STD = standard deviation. * indicates comparisons that fell outside the 2.5 to 97.5% percentile range of the bootstrapped distribution.

**Figure 3A** shows example HVS activity observed in rabbit-R (*n* = 15 sinusoidal and *n* = 2 microwire recording sites, second generation probe). This activity comprised high frequency spike-wave discharge, which was synchronous across all electrodes implanted on the same side of the brain, allowing accurate comparison of SNR across electrode types.

For this activity, the sinusoidal probes had higher mean peak-to-peak values compared to the microwire electrodes (**Figures 3B,F**) but also higher noise levels (**Figures 3C,F**). As a result there was no difference in the SNR between the two electrode types (**Figure 3D**). However, the SNR for the sinusoidal probe was more stable across the recording period, as revealed by a lower standard deviation across recording sessions (**Figure 3G**).

Overall impedance values were not significantly correlated with either mean peak-to-peak A (*R*^2^ = 0.037) or noise (*R*^2^ = 0.015) across all electrodes. However, impedance declined for both electrode types over the course of the implant period (**Figure 3E**). For the sinusoidal probe this resulted in improved SNR across the recording period (*R*^2^ = 0.76), an effect not seen for microwire electrodes (*R*^2^ = 0.01).

In summary, the sinusoidal electrode recorded higher mean peak-to-peak amplitude values with comparable stability to the microwire electrodes. Similar SNR values were obtained, however, the sinusoidal electrode had more stable SNR values across the recording period.

**FIGURE 4:**
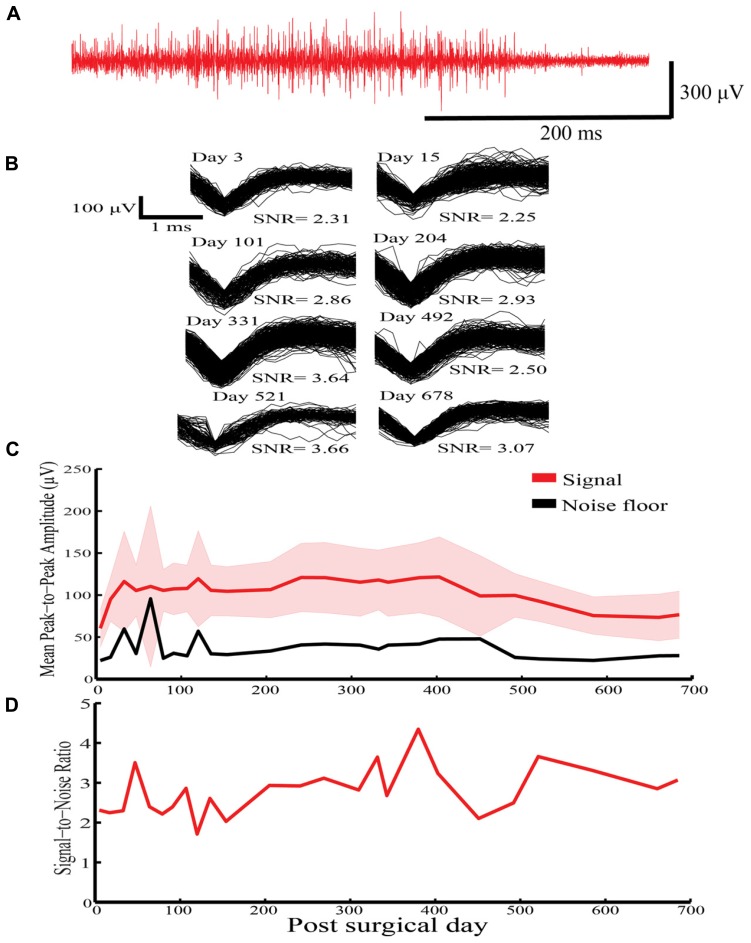
**High voltage spindle responses response recorded over a 678-indwelling period in Rabbit-N. (A)** Example recording trace for the HVS activity. **(B)** Overlain waveforms for selected days for the HVS response. **(C)** Daily mean peak-to-peak amplitude (±SEM) with corresponding noise floor recorded across the recording period for HVS activity. **(D)** Daily signal-to-noise ratio (SNR) values recorded as a function of indwelling period for HVS activity.

In Rabbit-N (first generation probe), HVS activity was recorded for a 678-day period on a solitary sinusoidal probe (**Figure 4A**). Similar waveforms were extracted over the 678-day indwelling period (**Figure 4B**). Further, stable mean peak-to-peak amplitude (**Figure 4C**) and SNR (**Figure 4D**) were recorded over the indwelling period. This initial result shows the potential for the sinusoidal electrode to record stable neuronal activity over chronic indwelling periods for >2 years.

### ELECTROPHYSIOLOGY: SINGLE-UNIT RECORDING EXAMPLE

**FIGURE 5:**
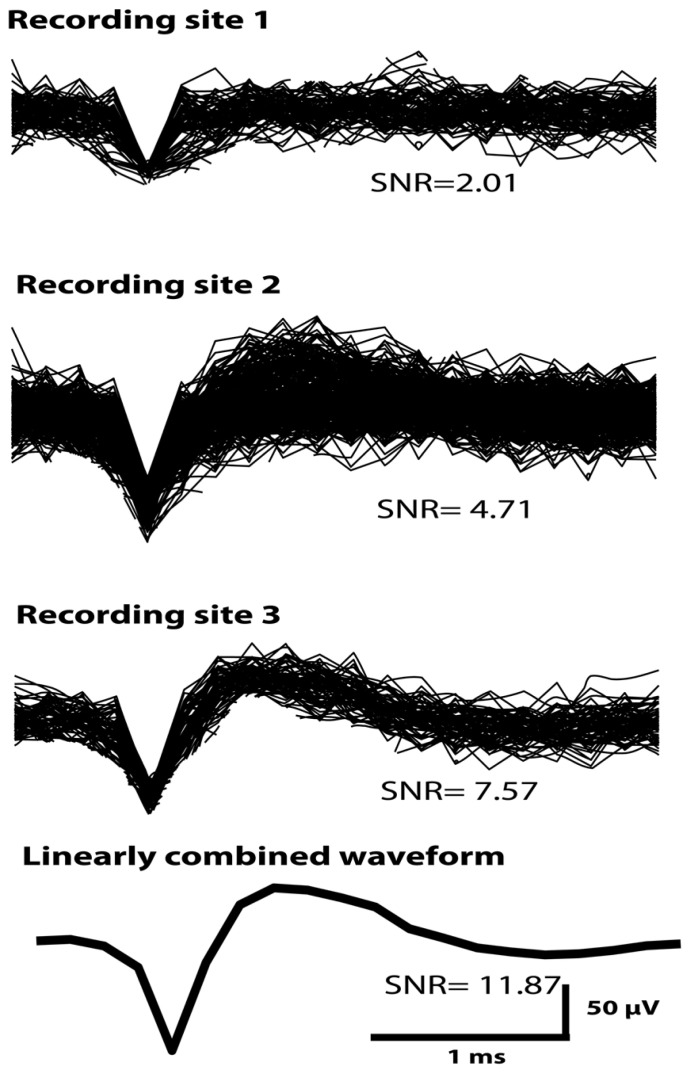
**A single unit recorded across all three sinusoidal recording sites in Rabbit-Lo.** An algorithm was used that optimized the SNR by forming a linear combination of the three available channels; the SNR of the combined waveform was 11.87. The SNR values from the individual recording sites were lower than this value.

**Figure 5** shows an example of a putative single unit obtained across all three electrode-recording sites of a sinusoidal electrode in Rabbit-Lo (second generation probe). The unit had different SNR and mean peak-to-peak amplitude on each contact, which allowed for effective unit isolation. Since the same action potential is observed on all three recordings, it is possible to improve SNR by linear combination. The three individual recording sites had SNR values of 2.01, 4.71, and 7.57. We used an algorithm, which chose the optimal linear combination to maximize the SNR; this achieved an SNR of 11.87. This example shows the utility of recording the same single unit across three electrode recording sites. An additional advantage of recording the same waveform on different electrodes is that differences in A may provide information additional to waveform shape that can be used for spike sorting (Gray et al., 1995; Harris et al., 2000).

### THE GLIAL RESPONSE TO IMPLANTED ELECTRODES

**FIGURE 6:**
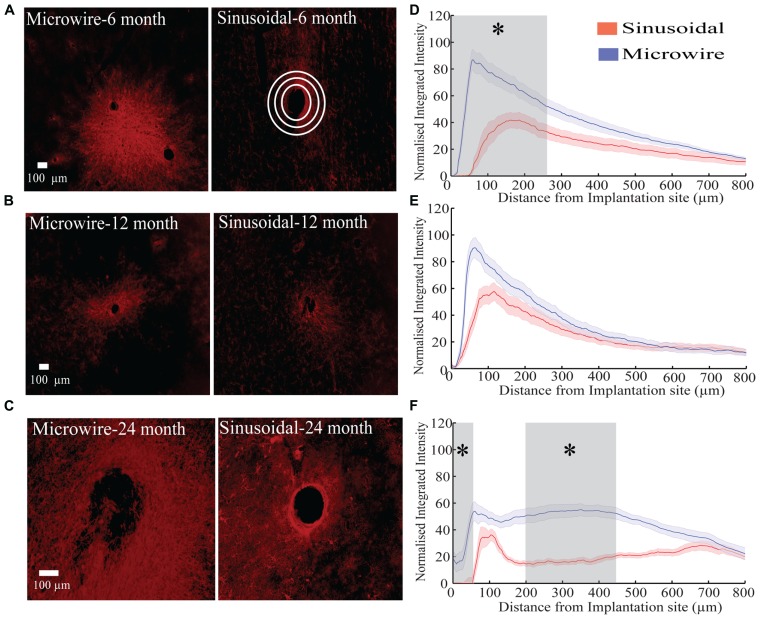
**The astrocytic response over a 6 (rabbit J, L), 12(rabbit P), and 24 (rabbit-N) month chronic indwelling period. (A)** Representative images for the astrocytic response for both electrode types at the 6 month time point. Note the interaction between the two microwire recording sites. White concentric rings show an illustrative example of measurements taken for the radial distribution intensity as a function of distance from the electrode implantation site. **(B)** Representative images for the astrocytic response for both electrode types at the 12 month time point. **(C)** Representative images for the astrocytic response for both electrode types at the 24 month time point. **(D)** Overall normalized integrated intensity response (±SEM) for both electrode types for the 6 month time point (*n* = 30 electrode tracts). Significant differences (gray shading) were found at 50–250 μm away from the electrode implantation site (*P* < 0.05). **(E)** Overall normalized integrated intensity response (±SEM) for both electrode types for the 12 month time point (*n* = 15 electrode tracts). No significant differences were found. **(F)** Overall normalized integrated intensity response (±SEM) for both electrode types for the 24 month time point (*n* = 15 electrode tracts). Significant differences were found at 50 and 150–450 μm away from the electrode implantation site (*P* < 0.05).

**FIGURE 7:**
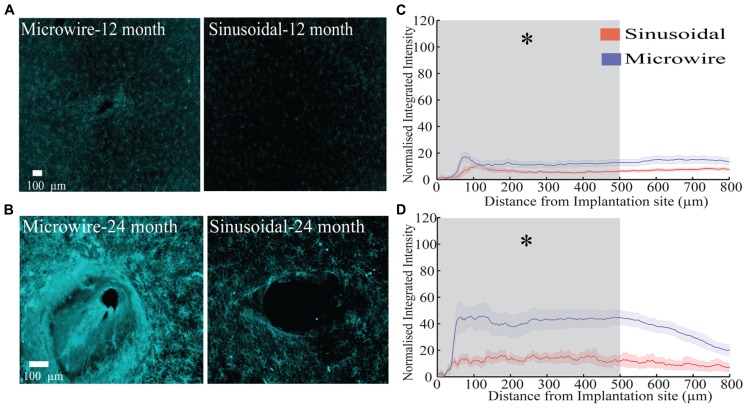
**The microglial response over a 12 (rabbit-P) and 24 (rabbit-N) month chronic indwelling period. (A)** Representative images for the microglial response for both electrode types at the 6 month time point. **(B)** Representative images for the microglial response for both electrode types at the 12 month time point. **(C)** Overall normalized integrated intensity response (±SEM) for both electrode types for the 12 month time point (*n* = 15 electrode tracts per electrode comparison). Significant differences were found at 50–500 μm away from the electrode implantation site (*P* < 0.05). **(D)** Overall normalized integrated intensity response (±SEM) for both electrode types for the 24 month time point (*n* = 15 electrode tracts per electrode comparison). For the bottom profile representation, significant differences were found at 50–500 μm away from the electrode implantation site (*P* < 0.05).

**FIGURE 8:**
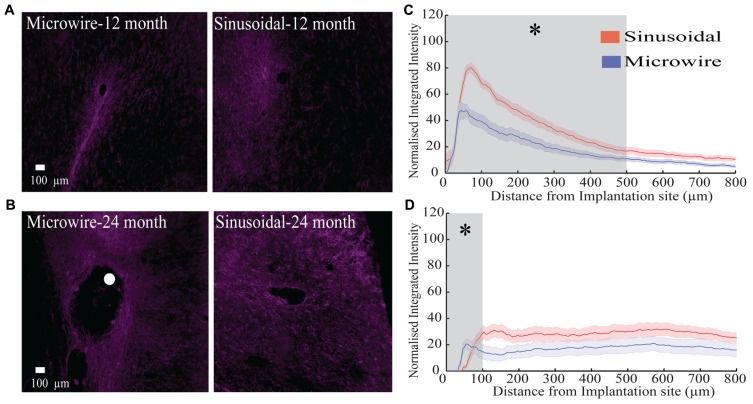
**The neurofilament response over a 12 (rabbit p) and 24 (rabbit-N) month chronic indwelling period. (A)** Representative images for the neurofilament response for both electrode types at the 6 month time point. **(B)** Representative images for the neurofilament response for both electrode types at the 12 month time point. The white dot represents the microwire electrode implantation site. **(C)** Overall normalized integrated intensity response (±SEM) for both electrode types for the 12 month time point (*n* = 15 electrode tracts per electrode comparison). Significant differences were found at 50–500 μm away from the electrode implantation site (*P* < 0.05). **(D)** Overall normalized integrated intensity response (±SEM) for both electrode types for the 24 month time point (*n* = 15 electrode tracts per electrode comparison). Significant differences were found at 50 and 100 μm away from the electrode implantation site (*P* < 0.05).

The glial response to both electrode types was assessed over a 6–24 month chronic indwelling period by staining for astrocytes (**Figure 6**), microglia (**Figure 7**) and neurofilament (**Figure 8**). Overall there was a decrease in the astrocytic response at both 6 (**Figures 6A,D**) and 24 months (**Figures 6C,F**) close to the sinusoidal probe implantation site and where the classical astrocytic response is usually found (Polikov et al., 2005). No difference was found between the two electrode types at 12 months (**Figures 6B,E**). We found a decrease in overall microgliosis over a region of 0–500 μm at both 12 (**Figures 7A,C**) and 24 months (**Figures 7B,D**). Interestingly, for the same time points we also found an increase in neurofilament staining around the sinusoidal probe (**Figure 8**). Therefore it appears that the sinusoidal electrodes were better integrated into the neural tissue with decreased gliosis compared to microwires.

## DISCUSSION

### MICROFABRICATION

Microfabrication was successful with high functional yields generated from a single wafer. As dilute TMAH was used to release devices, a clean room wet bench was not needed for device release. This is an advantage over other polymeric probes requiring strong acids or bases (Kohler et al., 2009; Kozai and Kipke, 2009; Wu et al., 2011) for a similar release process. In some previous work, polymeric probes have been successfully peeled off the silicon wafer substrate without the inclusion of a sacrificial layer (e.g., Takeuchi et al., 2004). We found a similar approach to be problematic during initial testing as some probes remained attached, decreasing the yield of finished probe. Inclusion of the sacrificial aluminum layer in our process aided device adhesion during fabrication, but allowed reliable and straightforward release. This led to high functional yields.

Adhesion between two parylene-C layers can be problematic. Multiple groups bake parylene-C post device fabrication to promote this adhesion, however, this is time consuming and risks damaging devices as temperatures (200°C) above the parylene-C glass transition are used (Pang et al., 2005; Metzen and Stieglitz, 2012; Kim et al., 2013; Kuo et al., 2013). Here we show that a slight RIE over-etch when etching the WTi metal layer has the benefit of surface roughening the first parylene-C layer, improving parylene adhesion and preventing subsequent device delamination.

Other groups have attempted to increase neuronal recordings longevity by decreasing the footprint of the probe (Lind et al., 2010; Yoshida Kozai et al., 2012). Using the equation for the deflection of a rectangular cantilever beam, which takes into account the material dimensions and YM, a decrease in dimensions will lead to a decrease in overall stiffness values (Lempka et al., 2006; Yoshida Kozai et al., 2012). Decreasing the material’s YM will have a similar effect. A limitation to decreasing probe dimensionality is the reduction in recording site area, which leads to higher obtained impedances (Ludwig et al., 2006). Electrodeposition of materials (e.g. PEDOT) onto the small recording site is required to achieve suitable impedance values for single-unit recording (Ludwig et al., 2011; Yoshida Kozai et al., 2012). However, these materials often have delamination issues >1 month post-implant (Yoshida Kozai et al., 2012).

The primary focus of this study was to compare the sinusoidal electrode to more conventional probes currently used for recording in non-human primates or humans. The improved performance over the rigid microwire electrode was largely due to reduced implant stiffness and reduced movement of the recording tip relative to the surrounding tissue. The contribution of the sinusoidal shaft to the success of the design is difficult to quantify from this study, as a straight-shafted control was not used. This comparison can be addressed in future studies both with a modeling approach and through *in vivo* implantation of a straight-shaft version of the probe.

### CHRONIC NEURONAL RECORDING

A striking result was the ability to record neuronal activity for >2 year period with stable recording parameters with the sinusoidal probe. Chronic recordings have also been obtained from other probes, such as the Utah array (>1–2 years; Rousche and Normann, 1998; Suner et al., 2005; Hochberg et al., 2006, 2012; Simeral et al., 2011; Collinger et al., 2012), Michigan probe (>6 weeks; Kipke et al., 2003; Vetter et al., 2004) and microwire probes (>1.5 years; Nicolelis et al., 2003; Jackson and Fetz, 2007). However, a distinct problem for all these probes is the lack of recording parameter stability. For microwires, Nicolelis et al. (2003) recorded activity from primate cortex. Based on the similarity of unsorted waveform clusters in principle component space, that study found 80% of the original units were still present after 2 days and 55% after 8 days (Nicolelis et al., 2003). Jackson and Fetz (2007) used the correlation coefficient between unsorted waveform averages and reported that 50% of original units were stable for a week, and 10% were stable through to 2 weeks. Dickey et al. (2009) used both average spike waveforms and interspike interval histograms to show that for a Utah array implanted in rhesus macaque monkeys 57, 43, and 39% of original units were stable for 7, 10, and 15 days respectively. In our hands, the sinusoidal probe provided more stable recordings judged from LFP and HVS activity than microwire electrodes. However, further testing is needed to corroborate this recording stability.

To compare our electrode performance with other commercially available electrodes a commonly used SNR measure was used. For our electrode, peak SNR values for HVS were between 3 and 6. An appropriate comparison is the SNR measured from other electrode designs using multi-unit activity. Jackson and Fetz (2007) obtained values around 7 for their microwire electrode. Ward et al. (2009) obtained values between 6 and 7 for multiple electrode types including the Utah array, microwire, and Michigan probe electrodes over a 30 day recording period. For novel carbon fiber electrodes, SNR values of 3–8 were obtained over a 5 week indwelling period (Yoshida Kozai et al., 2012). Our electrode had comparable or slightly lower SNR values, although variability in recording stability was less than the microwire electrode.

Signal amplitudes of 60–400 μV (Ward et al., 2009), 120–250 μV (Suner et al., 2005), 60–100 μV (Vetter et al., 2004), and 60–200 μV (Yoshida Kozai et al., 2012) have been obtained for microwire, Utah array, Michigan probe and carbon fiber electrodes for chronic recording. We show recording amplitude of 100–230 μV for HVS responses, which is comparable to these electrode types.

Noise values are rarely reported in chronic electrode studies, however, from the Michigan probe, a value of 6–13 μV has been described (Vetter et al., 2004). We here report noise values of 7–13 μV during HVS responses. This is comparable to the Michigan probe electrode.

Overall, the SNR for a recorded unit can be increased by capturing that single unit across all three sinusoidal probe-recording sites. In addition, being able to record the same neuron on different sites on the probe may help sorting spikes in multi-unit recordings, as is commonly done with tetrode recording (Tseng et al., 2011).

Measurements of electrode impedance were not correlated with noise or signal values. For saline impedance testing, thermal noise is the main component of noise as there is no neuronal activity present. *In vivo*, SNR should follow impedance trends as thermal noise is directly related to the electrode impedance (Ludwig et al., 2006). As there was no correlation, it is possible that other sources of noise (e.g., biological or instrumentation noise) may have dominated the overall noise floor, as sources of noise summate in quadrature. Other groups have reported this phenomenon: SNR measurements are not related to 1 kHz *in vivo* impedance in both rodents and non-human primates for a variety of microelectrodes (Suner et al., 2005; Ward et al., 2009; Kim et al., 2013). Therefore, long-term impedance measurements may not be the best indicator for understanding chronic electrode performance.

Overall, our electrode had stable or initially declining impedances that stabilized over the indwelling period, similar to the microwire electrode. In contrast, silicon based probes have increasing impedances over both a 30 (Ward et al., 2009) and 81(Vetter et al., 2004) day indwelling period. For the Utah array there is a drop in impedance >1 year post-implant, which is related to electrode degradation (Suner et al., 2005). The drop in microwire impedance is related to the gradual insulation delamination surrounding the recording site, which increases the geometric surface area (GSA) available for recording (Patrick et al., 2011; Prasad et al., 2012).

For HVS responses, a few sinusoidal electrodes had drops in impedance after >20 days post-surgery, leading to improved recording characteristics. This may be related to the polyimide ball anchor. Gradual polyimide ball dissolution may have resulted in an increase in available GSA for recording. An alternative anchor which is more stable may need to be considered for future studies.

### GLIAL RESPONSE

In this study, the sinusoidal probe was compared to microwire electrodes. Although the electrode was not compared to standard silicon probes, an emergent hierarchy is evident from the literature. In general silicon probes give a heightened immune response in relation to microwire style probes (Winslow et al., 2010; Yoshida Kozai et al., 2012). As we show decreased overall gliosis at multiple time points, it is reasonable to assume that we should also have a decreased response when comparing to standard silicon probes.

The sinusoidal probe had decreased overall gliosis measured by astrocytosis for the 6 month time point and by both astrocytosis and microgliosis for the 12 and 24 month time point. There was an increase in neurofilament staining around the sinusoidal probe at the 12 and 24 month time point. It appears that electrode designs incorporating micromotion-reducing measures can reduce gliosis and promote neuronal integration. Interestingly, we show an increase in neurofilament staining without the use of neurotropic factors, such as that used in the cone electrode (Kennedy, 1989; Kennedy et al., 1992; Bartels et al., 2008). The addition of a three dimension ball anchor seemed to attract neurites similar to the effect observed by Winslow and Tresco (2010) with cylindrical microwire electrodes over a 12-week indwelling period. The decreased microglial response is most likely the cause for the better neuronal integration seen with neurofilament staining for the sinusoidal probe. A heightened microglial response is associated with the loss of neurons in the immediate vicinity of the probe (~100 μm away), designated the “kill zone” (Biran et al., 2007); this is also the maximum theorized distance over which an implanted neuronal probe can record neuronal activity (Polikov et al., 2005). Therefore, an advantage of the sinusoidal probe is a decreased microgliosis response over a chronic indwelling period that limits the “kill zone” region.

Intriguingly individual microwire electrode tracts can interact with one another, producing interacting tracks of glial scarring over chronic indwelling period of >6 months. This is not ideal, as interaction between two electrode tracts may induce biological noise and alter overall network effects for the specific recording cells of interest as astrocytes can interact with neurons (Giaume et al., 2010; Gourine et al., 2010; Sasaki et al., 2012).

There are reports that at long times post-implant, gliosis extends beyond a reported distance of 500 μm (Biran et al., 2005; Polikov et al., 2005). This raises the possibility that on-going gliosis could continue to spread far beyond the original implantation site, affecting considerable volumes of brain tissue. Such chronic spreading gliosis needs to be prevented to maintain stable recording parameters, especially in the presence of multiple implants.

### FUTURE DIRECTIONS

Although the results of this initial study are promising, further testing is needed to corroborate the effectiveness of the sinusoidal probe as a chronic implant. Testing is needed in larger animals (e.g., non-human primates) where the problem of micromotion is far greater due to different patterns of animal behavior and skull sizes. We have verified that our insertion technique is suitable for non-human primates (data not shown), so this translational step beyond rodents and lagomorphs is a possibility.

### CONCLUSION

To our knowledge this is the first study to show the usefulness of addressing micromotion-induced trauma with flexible electrodes more than 6 months after implant. We have shown that the sinusoidal probe appeared to be more stable in recording parameters over a 6–24 month chronic indwelling period in terms of LFP and HVS, especially in terms of LFP power and SNR. Further, we show that it is possible to register neuronal activity beyond 2-years post-implant with the sinusoidal probe. Gliosis was reduced at the recording tip of the sinusoidal compared to the rigid microwire electrodes at 6, 12, and 24-month time points, and there was better neuronal integration at 12 and 24 months. However, further studies are needed to corroborate these initial promising results. This initial study shows that the sinusoidal probe is a potential alternative to silicon, microwire and other polymeric probes for chronic neuronal recording.

## Conflict of Interest Statement

The authors declare that the research was conducted in the absence of any commercial or financial relationships that could be construed as a potential conflict of interest.
